# Neuronal Transdifferentiation Potential of Human Mesenchymal Stem Cells from Neonatal and Adult Sources by a Small Molecule Cocktail

**DOI:** 10.1155/2019/7627148

**Published:** 2019-04-01

**Authors:** Lorena V. Cortés-Medina, Herminia Pasantes-Morales, Alejandro Aguilera-Castrejon, Arturo Picones, Cesar O. Lara-Figueroa, Enoch Luis, Juan Jose Montesinos, Victor A. Cortés-Morales, M. P. De la Rosa Ruiz, Erika Hernández-Estévez, Laura C. Bonifaz, Marco Antonio Alvarez-Perez, Gerardo Ramos-Mandujano

**Affiliations:** ^1^División de Neurociencias, Instituto de Fisiología Celular, Universidad Nacional Autónoma de México, Mexico City 04510, Mexico; ^2^Department of Molecular Genetics, Weizmann Institute of Science, Rehovot 76100, Israel; ^3^Laboratorio Nacional de Canalopatías, Instituto de Fisiología Celular, Universidad Nacional Autónoma de México, Mexico City 04510, Mexico; ^4^Cátedras CONACyT Instituto de Fisiología Celular, Universidad Nacional Autónoma de México, Mexico City 04510, Mexico; ^5^Mesenchymal Stem Cells Laboratory, Oncology Research Unit, Oncology Hospital, National Medical Center, IMSS, Mexico City 06720, Mexico; ^6^Unidad de Investigación Médica en Inmunoquímica, Hospital de Especialidades, Centro Médico Nacional Siglo XXI, IMSS, Mexico City 06720, Mexico; ^7^Tissue Bioengineering Laboratory, Division of Graduate Studies and Research of the Faculty of Dentistry, UNAM, Mexico City 04510, Mexico

## Abstract

Human mesenchymal stem cells (MSCs) are good candidates for brain cell replacement strategies and have already been used as adjuvant treatments in neurological disorders. MSCs can be obtained from many different sources, and the present study compares the potential of neuronal transdifferentiation in MSCs from adult and neonatal sources (Wharton's jelly (WhJ), dental pulp (DP), periodontal ligament (PDL), gingival tissue (GT), dermis (SK), placenta (PLAC), and umbilical cord blood (UCB)) with a protocol previously tested in bone marrow- (BM-) MSCs consisting of a cocktail of six small molecules: I-BET151, CHIR99021, forskolin, RepSox, Y-27632, and dbcAMP (ICFRYA). Neuronal morphology and the presence of cells positive for neuronal markers (TUJ1 and MAP2) were considered attributes of neuronal induction. The ICFRYA cocktail did not induce neuronal features in WhJ-MSCs, and these features were only partial in the MSCs from dental tissues, SK-MSCs, and PLAC-MSCs. The best response was found in UCB-MSCs, which was comparable to the response of BM-MSCs. The addition of neurotrophic factors to the ICFRYA cocktail significantly increased the number of cells with complex neuron-like morphology and increased the number of cells positive for mature neuronal markers in BM- and UCB-MSCs. The neuronal cells generated from UCB-MSCs and BM-MSCs showed increased reactivity of the neuronal genes TUJ1, MAP2, NF-H, NCAM, ND1, TAU, ENO2, GABA, and NeuN as well as down- and upregulation of MSC and neuronal genes, respectively. The present study showed marked differences between the MSCs from different sources in response to the transdifferentiation protocol used here. These results may contribute to identifying the best source of MSCs for potential cell replacement therapies.

## 1. Introduction

The *in vitro* generation of neuronal cells from neural (NSCs), embryonic (ESCs), and induced pluripotent stem cells (iPSCs), or by neuronal transdifferentiation of somatic cells by transcription factors (TF) has emerged as a useful strategy for cell replacement therapies in neurological disorders [1–3]; however, technical limitations, graft rejection, ethical issues, and/or tumorigenic risk are associated with the neurons derived from such processes [4–6]. Therefore, recent efforts have been focused on finding more suitable cell types or avoiding genetic manipulation for the generation of neurons [4, 7–11]. In this respect, mesenchymal stem cells (MSCs) offer some advantages over other cell types. MSCs are potentially able to differentiate into various cell lineages (including neurons), are easy to isolate and expand, have a low tumorigenic risk and low grafting rejection, and lack ethical issues [12–15]. These properties point to MSCs as suitable sources for cell replacement therapy in neurological disorders [16–19]; however, an optimal protocol to induce their conversion into neurons remains unestablished.

Chemical compounds known as small molecules have been shown to replace exogenous TF during cell reprogramming [7–9, 11]. Recent reports demonstrated the neuronal transdifferentiation of fibroblasts and astrocytes by small molecule cocktails [20–23]. These molecules act by modulating signaling pathways and epigenetic mechanisms implicated in cell reprogramming, neuronal specification, or neuronal survival [21], representing a convenient strategy to avoid the risks of genetic manipulation in the generation of induced neurons. In our previous report, after a small molecule screening assay, we found that a cocktail containing I-BET151, CHIR99021, forskolin, RepSox, Y-27632, and cAMP (ICFRYA) induced the formation of cells with neuron-like morphology and positive for TUJ1 and MAP2 from bone marrow- (BM-) MSCs [10].

MSCs can be isolated from many adult and neonatal tissues. However, comparative studies indicate that the MSCs from different tissues present differences in the efficiency of trilineage differentiation and other functional abilities, even though they meet the properties to be considered MSCs [24–27]. The present study is aimed at comparing the neuronal transdifferentiation potential of adult and neonatal MSCs obtained from different sources. To this end, we evaluated the neuronal-like morphology and neuronal markers induced by the ICFRYA cocktail in MSCs obtained from bone marrow (BM), skin (SK), dental pulp (DP), periodontal ligament (PDL), gingival tissue (GT), Wharton jelly (WhJ), placenta (PLAC), and umbilical cord blood (UCB). Neuronal induction was successful in the MSCs from some but not all sources. Strategies were selected to improve the induction of the MSC sources that showed neuronal properties. The presence of mature neuron markers, changes in global gene expression, and electrophysiological activity were examined in cells in which neuronal transdifferentiation was presumed.

## 2. Materials and Methods

### 2.1. Reagents and Antibodies

Neurobasal medium, *α*-MEM, RPMI medium, DMEM low glucose (DMEM-lg), DMEM/F-12, fetal bovine serum (FBS), L-glutamine, dispase II, Glutamax, antibiotics, trypsin/EDTA, human neurotrophic factors, N2, and B27 were all purchased from Gibco™ Thermo Fisher Scientific Inc. (MA, USA). Fibronectin, heparin, gelatin, nonessential amino acids, Hoechst 33258, and normal goat serum (NGT) were acquired from Sigma-Aldrich, Merck (St. Louis, MO, USA). The following primary antibodies were used: mouse anti-TUJ1 (SC-80016) and rabbit anti-MAP2 (SC-20172) obtained from Santa Cruz Biotechnology (CA, USA); rabbit anti-GABAB (SC-376282), ENO2 (GTX113428), TAU (GTX116044), and mouse anti-NF-H (GTX27795) from GeneTex Inc. (CA, USA); and rabbit anti-NeuN (MAB377) from Merck-Millipore (Darmstadt, Germany). Secondary antibodies against goat anti-mouse IgG conjugated to Alexa Fluor 488 and goat anti-rabbit IgG conjugated to Alexa Fluor 568 were obtained from Molecular Probes, Thermo Fisher Scientific Inc. (MA, USA). Small molecules I-BET151, CHIR99021, forskolin, RepSox, Y-27632, and dibutyryl cAMP were purchased from Sigma-Aldrich, Merck (St. Louis, MO, USA).

### 2.2. MSC Isolation and Characterization

All samples were isolated from healthy donors according to the Declaration of Helsinki. The bone marrow samples were obtained according to the Local Ethics Committee of the Villa Coapa Hospital, Mexican Institute for Social Security (IMSS, Mexico). The umbilical cord blood and placenta samples were collected according to the Local Ethics Committee of the Troncoso Hospital (IMSS, Mexico). The dental tissue samples were obtained according to the local Ethics Committee of the Orthodontics Clinic and the Faculty of Dentistry, National Autonomous University of Mexico. The skin samples were collected according to the Local Ethics Committee of the Dermatology Hospital, Health Secretary, Mexico City (SS, Mexico).

Mononuclear cells were isolated from bone marrow and umbilical cord blood samples by a Ficoll gradient, and cells from the internal area of the central placenta lobules were obtained using an enzymatic digestion procedure (Trypsin-EDTA). These cell samples were cultured in DMEM-lg plus 4 mM L-glutamine, 50 U/mL of penicillin, 50 *μ*g/mL of streptomycin, and 50 *μ*g/mL of gentamicin (DMEM-lg) and supplemented with FBS-10% [28]. The Wharton's jelly (membrane), gingival tissue, periodontal ligament, and dental pulp tissue samples were cut into small pieces and cultured as explants in *α*-MEM supplemented with FBS-15%. Finally, the skin samples were placed overnight in RPMI medium and dispase II, and then, the dermis was mechanically separated from the epidermis. Cells from the dermis were isolated by allowing them to migrate from the dermal segments placed in culture (DMEM-lg supplemented with FBS-10%). For all samples, the medium was changed every two days. The adherent cells were subcultured using trypsin/EDTA at 2 × 10^2^ cells/cm^2^ in DMEM-lg supplemented with FBS-10%. All experiments were performed with MSCs between the fourth and sixth passages.

MSCs were characterized based on morphological, phenotypic, and differentiation parameters [28, 29]. FITC, PE, or APC-conjugated monoclonal antibodies against CD73, CD90, CD45 (BD Biosciences, San Diego, CA, USA), CD105, CD13, CD14, (Caltag, Buckingham, UK), HLA-ABC, HLA-DR, CD31, and CD34 (Invitrogen, Carlsbad, CA, USA) were used for immunophenotypic characterizations and analyzed on a FACSCanto II flow cytometer (BD Biosciences, Fullerton CA, USA). Adipogenic differentiation was determined by visualizing the presence of Oil Red O-stained (Sigma-Aldrich, Merck) lipid vacuoles. Osteogenic differentiation was assessed by alkaline phosphatase staining (FAST BCIP/NBT; Sigma-Aldrich, Merck). Chondrogenic differentiation was induced over 28 days with 10 ng/mL TGF-*β* (PeproTech). The resulting micromasses were fixed, embedded, and sliced, and cross-sections were stained with Alcian blue dye (Sigma-Aldrich, Merck).

### 2.3. Neuronal Induction by the ICFRYA Cocktail

Adult and neonatal MSCs were seeded onto fibronectin (2 *μ*g/cm^2^)/0.1% gelatin-coated plates at a density of 2 × 10^4^ cells/cm^2^ and cultured in MSC growth medium for one day. For chemical induction, the cells were washed with serum-free medium and then cultured with neuronal induction medium (50% neurobasal medium, 50% DMEM/F12 with 1 × N2 and 1 × B27 with vitamin A, 1 × Glutamax, 1 × nonessential amino acids, and 20 ng/mL FGF2 plus 5 *μ*g/mL heparin) plus the defined chemical cocktails for 8 or 4 days (as appropriate) at 37°C and 5% CO_2_. The concentrations of the molecules in the ICFRYA cocktail were 1 *μ*M I-BET151, 20 *μ*M CHIR99021, 50 *μ*M forskolin, 1 *μ*M RepSox, 5 *μ*M Y-27632, and 100 *μ*M dbcAMP [10]. The neuronal induction medium was replaced on day 4. When indicated, the neuronal induction medium plus the ICFRYA cocktail was supplemented with human brain-derived neurotrophic factor (hBDNF), human glial cell-derived neurotrophic factor (hGDNF), and neurotrophin-3 (NT-3) at 20 ng/mL each.

### 2.4. In Situ Analysis of Cell Viability

Cells were incubated for 10 min with 1 *μ*g/mL propidium iodide. Microphotographs were obtained using a direct epifluorescence microscope Olympus 1X71 with 10x magnification and the QCapture Pro 6.0 software.

### 2.5. Immunocytochemistry

After culture, the cells were fixed/permeabilized with cold methanol for 10 min and washed three times with PBS. The cells were blocked using PBS/0.1% BSA + 10% GS (goat serum) for 1 h. Then, the cells were incubated overnight at 4°C with primary antibodies: mouse anti-TUJ1 (1 : 1000), anti-NF-H (1 : 250), and anti-NeuN (1 : 200) and rabbit anti-MAP2 (1 : 500), anti-TAU (1 : 1000), anti-GABAB (1 : 250), and anti-ENO2 (1 : 250). Then, the cells were incubated for 1 h with goat anti-mouse Alexa 488 or goat anti-rabbit Alexa 568 as appropriate. The nuclei were counterstained with 1 *μ*g/mL Hoechst 33258 diluted in PBS. Microphotographs were obtained by the direct epifluorescence microscope Olympus IX71 using the QCapture Pro 6.0 software, and data were analyzed with ImageJ software. At least 500 nuclei from five randomly selected fields were counted to calculate the percentage of positive cells.

### 2.6. Real-Time Quantitative PCR (qPCR)

RNA samples from cultures were extracted with the GenElute Mammalian Total RNA Miniprep Kit (RTN70 Sigma-Aldrich, St. Louis, MO, USA) according to the manufacturer's instructions. The concentration of RNA samples was quantified by a NanoDrop spectrophotometer (Thermo Scientific). Complementary DNA (cDNA) was synthesized from 1 *μ*g RNA with the iScript cDNA Synthesis Kit (Bio-Rad Laboratories Inc., CA, USA) and a Veriti® 96-Well Thermal Cycler (Applied Biosystems). PCR primers were designed using the last reference sequence (RefSeq) version of each gene with the PrimerBlast software; Supplementary Table 5 includes a list of primers used in this study. The qPCR reactions were conducted using a QuantiFast SYBR Green PCR Kit (Qiagen, MD, USA), according to the manufacturer's instructions, and run on a StepOnePlus Real-Time PCR system (Applied Biosystems). After amplification, the melting curves of the RT-PCR products were determined to demonstrate product specificity. PCR efficiency was optimal and ranged from 90% to 100% in the different target gene qPCR assays. For the selection of the best reference gene, a comparison of the transcriptional variation of different housekeeping genes in response to the conditions was performed. The gene with the least CT variation between treatments and control cultures was selected. Thus, gene expression was normalized to TATA-Box binding protein (TBP) mRNA. The relative quantification of mRNA levels was performed using the Pfaffl method [30].

### 2.7. Microarray Assay

Cells were incubated as described in the neuronal maturation protocol. The samples were then processed for RNA extraction. Total RNA (10 *μ*g) was used for cDNA synthesis incorporating dUTP-Alexa 555 or dUTP-Alexa 647 and employing the CyScribe First-Strand cDNA labeling kit (Amersham). Equal quantities of labeled cDNA were hybridized using the hybridization solution HybIT2 (TeleChem International Inc.) on the collection of human arrays (10 K) for 14 h at 42°C. The acquisition and quantification of array images were performed in the ScanArray 4000 with its accompanying software from Packard BioChips. For each spot, the mean density, mean background, and mean normalized signal values were calculated with the Array-Pro Analyzer software from Media Cybernetics. Microarray data analysis was performed with the free software genArise, which was developed in the Computing Unit of the UNAM Cellular Physiology Institute (http://www.ifc.unam.mx/genarise/). The microarray data were deposited in the Gene Expression Omnibus database repository (GSE120681). The functional annotation gene analysis analyzed the up- and downregulated genes derived from the microarray assay using DAVID 2.0 (https://david.ncifcrf.gov/tools.jsp) and bioinformatics resources to obtain the Gene Ontology functional annotation classification of the genes.

### 2.8. Electrophysiological Activity Recording

Whole-cell ion currents were experimentally recorded with an arrangement of Axopatch 200B/Digidata 1550/pCamp10 (amplifier/analog-digital converter/software, all from Molecular Devices, Sunnyvale, CA), analog filtered at 5 kHz and digitally sampled at 10 kHz. Patch-clamp pipettes were made of borosilicate glass (World Precision Instrument) using a P-97 puller (Sutter Instruments, Novato, CA). The intracellular pipette filling solution was composed of (in mM) 10 NaCl, 40 KCl, 10 HEPES, 5 EGTA, 3 MgCl_2_, 95 K-gluconate, and 10 glucose (pH 7.2 adjusted with KOH). The extracellular solution contained (in mM) 140 NaCl, 4 KCl, 1 MgCl_2_, 2 CaCl_2_, 10 HEPES, and 5 glucose (pH 7.4 adjusted with NaOH). All solutions were perfused using a custom-made gravity-based perfusion system. The voltage-clamp protocol used in all experiments was 200 ms step pulses from -120 to +50 mV in 10 mV increments from a holding voltage of -70 mV.

### 2.9. Statistical Analysis

The results were expressed as the mean ± standard error (SEM) from three independent experiments of three biological samples or 4-6 independent experiments from one biological sample as indicated. The statistical analyses were performed using Student's *t*-test for between-group comparisons and a one-way ANOVA and the Tukey test for multiple comparisons. GraphPad Prism software for Windows version 6 (La Jolla, CA, USA) was used for all statistical procedures. Differences with *p* < 0.05 were considered statistically significant.

## 3. Results

### 3.1. MSC Characterization

**Figure 1:**
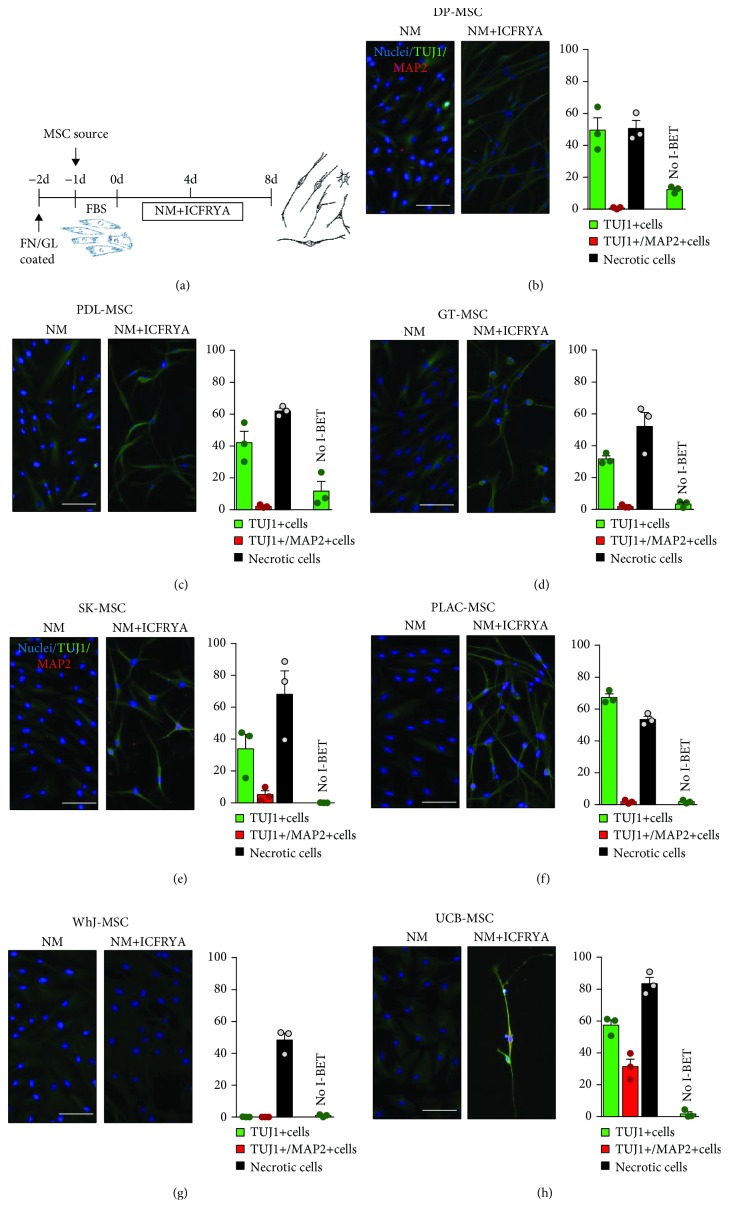
Neuronal properties induced by the ICFRYA small molecule cocktail in different sources of human mesenchymal stem cells (hMSCs). (a) Schematic diagram of the neuronal induction protocol. One day prior to the neuronal induction, MSCs were seeded on FN/GL-treated plates, and the medium was replaced with neuronal medium (NM: N2/B27/FGF/EGF) or NM containing the ICFRYA small molecule cocktail: I: I-BET151; C: CHIR99021; F: forskolin; R: RepSox; Y: Y27632; A: cAMP. NM or NM plus the ICFRYA cocktail was changed at day 4, and the neuronal properties and necrosis were evaluated on day 8. (b–h) Representative images and percentages of neuron-like morphology cells positive for TUJ1 (green bars), TUJ1/MAP2 (red bars), and necrotic cells (black bars). MSCs cultured with NM maintained fibroblastic morphology (panels (b–h) left picture) and acquired neuron-like morphology only under small molecule stimulation (panels (b–h) right picture). The presence of neuronal markers was estimated by immunostaining, and necrosis was evaluated in situ using propidium iodide (PI). In addition to marker reactivity, neuronal-like morphology was considered to define TUJ1^+^ cells and TUJ1^+^/MAP2^+^ cells. The data are presented as the mean ± SEM of *n* = 8 from three biological replicates in three independent experiments. Scale bars represent 100 *μ*m. FN/GL: fibronectin-gelatin; FBS: fetal bovine serum; NM: neuronal medium; ICFRYA: I-BET151, CHIR99021, forskolin, RepSox, Y27632, cAMP.

Mesenchymal stem cells (MSCs) were isolated from human adult or neonatal sources (Supplementary Table 1) and characterized according to the criteria defining human MSCs proposed by the International Society for Cellular Therapy [31]. For all MSC samples, the cells adhered to the plastic exhibited fibroblastic morphology (Supplementary Figure 1), were positive for MSC markers (CD105, CD90, and CD73), were negative for hematopoietic markers and HLA-DR surface molecules (Supplementary Table 2), and were capable of differentiating into adipocytes, osteoblasts, and chondrocytes (Supplementary Table 3). The neuronal medium per se (NM: N2/B27/hFGF) did not induce neuronal morphology or reactivity to neuronal markers (Supplementary Figure 3); however, a weak reactivity against TUJ1 was observed in all MSC sources (Figures 1(b)–1(h) left picture).

### 3.2. Neuronal Induction by the ICFRYA Cocktail of Adult and Neonatal MSC Sources

**Figure 2:**
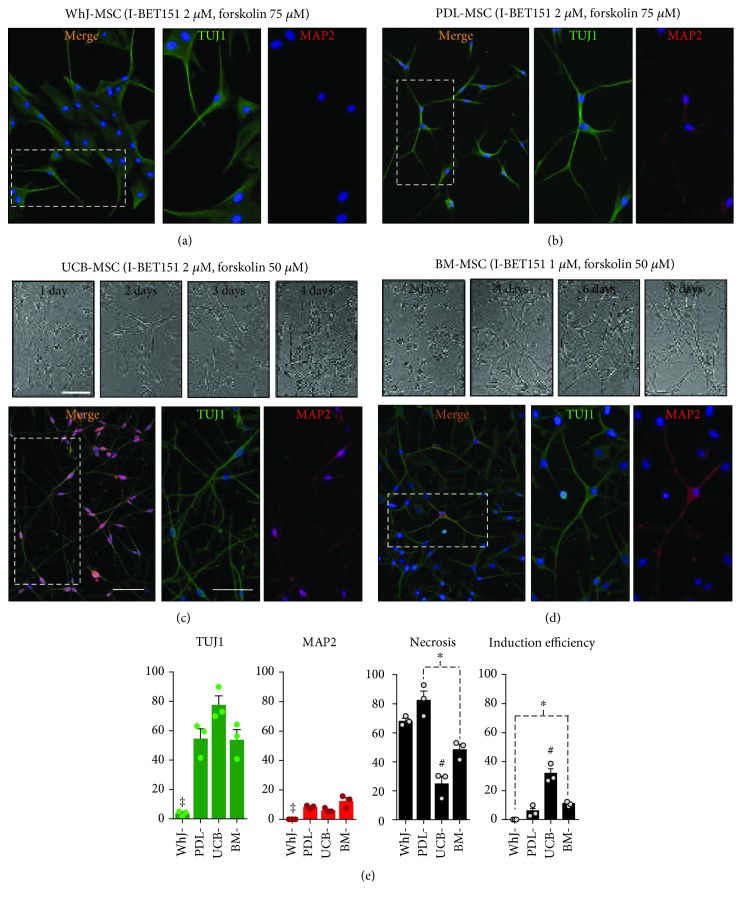
Effect of increasing I-BET151 and/or forskolin concentration on neuronal induction. Neuronal induction was conducted as in Figure 1, but the I-BET151 concentration was increased from 1 *μ*M to 2 *μ*M and/or forskolin concentration was increased from 50 *μ*M to 75 *μ*M. (a) Wharton's jelly cells did not show neuronal induction. (b) PDL-MSCs are shown as the representative source of an intermediate response (Supplementary Table 4), and (c, d) UCB and BM-MSCs show highly efficient neuronal induction. The morphological changes throughout culturing are shown in bright field pictures. UCB acquired neuron-like morphology after 2 days of induction, developing complex neuronal morphology at 4 days, while BM developed similar morphology after 8 days. Immunostaining shows that UCB- and BM-MSC developed complex TUJ1^+^ and MAP2^+^ neurite-like outgrowths. (e) Quantification of TUJ1^+^ cells, MAP2^+^ cells, and necrotic cells as in Figure 1. Induction efficiencies were calculated as the percentage of induced TUJ1^+^ neuronal cells versus initial cell number at day 0. The data are presented as the mean ± SEM. Significant differences were determined using ANOVA and Tukey's test. *p* < 0.05: ^‡^WhJ-MSCs or ^#^UCB-MSCs vs. the other sources, and ^∗^ *p* < 0.05 between sources indicated with dotted lines. *n* = 8 from three independent biological replicates. Scale bars represent 100 *μ*m.

Neuronal induction of all MSC sources was conducted using the ICFRYA small molecule cocktail protocol (Figure 1(a)) as previously reported for BM-MSCs [10]. In addition to neuronal properties, necrotic cell death was evaluated by propidium iodide staining. The neuronal induction effects of the ICFRYA cocktail on MSCs from different sources varied. Neuron-like cells with reactivity to TUJ1 were induced in DP-MSCs (50 ± 7.7%), PDL-MSCs (42 ± 7.1%), GT-MSCs (32 ± 1.9%), SK-MSCs (34 ± 9.1%), and PLAC-MSCs (67 ± 2.3%); however, very low percentages of MAP2^+^ cells were found (0.7% to 5.9%) (Figures 1(b)–1(f), right pictures and graphs). In these MSC sources, the cocktail produced 48%-68% necrosis. In WhJ-MSCs, the chemical cocktail did not evoke cells with neuronal properties, but high levels of necrosis were present (Figure 1(g) right picture and graph). In UCB-MSCs, the ICFRYA cocktail induced neuronal properties, i.e., bipolar morphology with a high percentage of TUJ1^+^ cells (57 ± 3.3%) and coexpression of MAP2 in 27 ± 3.9%; however, a very high rate of necrosis (84 ± 6.8%) was observed (Figure 1(h) right picture and graph). The removal of I-BET151 from the chemical cocktail reduced the necrotic cell death strongly in all MSC sources and also reduced the expression of neuronal properties (Figures 1(b)–1(h), no I-BET bars; Supplementary Table 4). It is noteworthy that I-BET alone did not exhibit any effects on the MSCs from any tissue (data not shown). I-BET151 and forskolin are considered key molecules for chemical-based neuronal transdifferentiation; in other reports, high concentrations of these molecules have been used for neuronal transdifferentiation [22]. However, increasing the concentration of I-BET151 (1 *μ*M to 2 *μ*M) and/or forskolin (50 *μ*M to 75 *μ*M) did not improve the expression of neuronal properties on WhJ-, DP-, PDL-, GT-, SK-, or PLAC-MSCs but did increase necrosis (Supplementary Table 4 and Supplementary Figure 2). As illustrated in Figures 2(a) and 2(e), very few cells from WhJ-MSCs presented a bipolar morphology and reactivity to TUJ1. In PDL-MSCs, although MAP2 reactivity was increased, the signal was not associated with cell protrusions, and almost all cells maintained the bipolar morphology (Figures 2(b) and (e)). On the other hand, in UCB-MSCs incubated with ICFRYA containing 2 *μ*M I-BET151, neuronal morphology was observed after two days of induction (Figure 2(c)), notably reducing the induction time from 8 to 4 days. At this time, UCB-MSCs exhibited proper neuron-like induction with 77.6 ± 10.7% TUJ1^+^ cells, protrusions with secondary ramifications, MAP2 reactivity associated with the branches, and only 25% necrotic death (Figures 2(c) and 2(d) and Supplementary Table 4). In our previous report, the ICFRYA cocktail containing 1 *μ*M I-BET and 50 *μ*M forskolin induced neuron-like cells from BM-MSCs with reactivity to TUJ1 and MAP2. BM-MSCs were added as the gold standard to compare neuronal induction with the other sources. Using different concentrations of the molecules, neuronal morphology was evident after 4 days (Figure 2(d)), and the original ICFRYA molecule concentration showed the best induction in BM-MSCs (Figures 2(d) and 2(e) and Supplementary Table 4).

### 3.3. Neurotrophic Factors Improve Neuronal Induction of BM- and UCB-MSCs by ICFRYA

**Figure 3:**
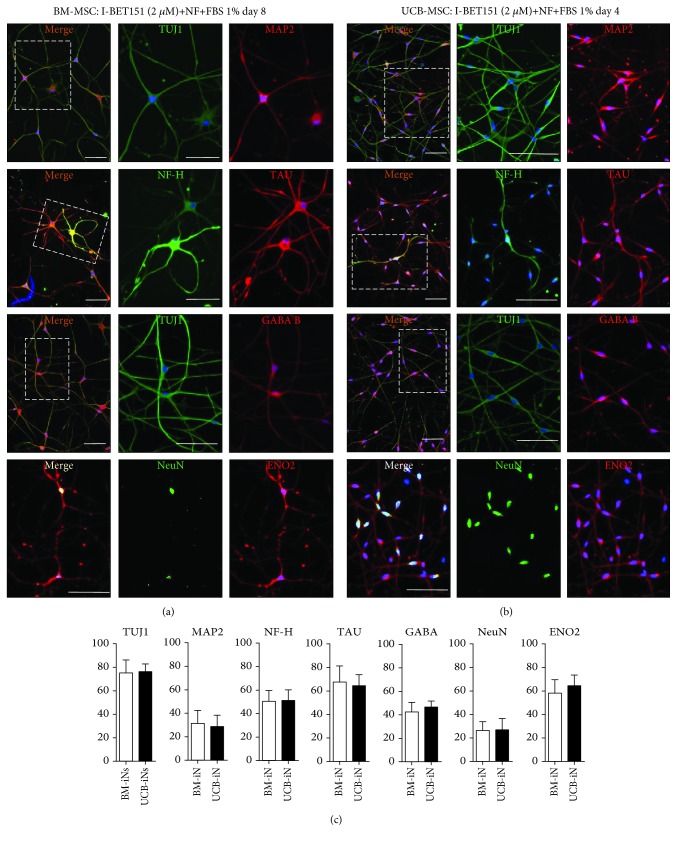
Presence of mature neuronal markers after induction with ICFRYA plus neurotrophic factors and FBS in UCB- and BM-MSCs. (a, b) Representative pictures of TUJ1, MAP2, TAU, NF-H, GABA B, ENO2, and NeuN positive cells after neuronal induction. The images on A show the merge, and the images on B and C show the individual signal for each antibody. (a) BM-MSCs after 8 days of induction and (b) UCB-MSCs after 4 days of induction. (c) Quantification of cells positive for the neuronal markers induced by the ICFRYA cocktail plus neurotrophic factors. The data are presented as the mean ± SEM (^∗^ *p* < 0.05, Student's *t*-test) of *n* = 8 from three independent experiments. Scale bars represent 100 *μ*m.

To examine a possible effect of neurotrophic factors on the improvement of neuronal induction by the ICFRYA cocktail, BM- and UCB-MSCs were cocultured with astrocytes and/or in the presence of neurotrophic factors (BDNF, GDNF, and neurotrophin-3) and fetal bovine serum (FBS) 1%. Coculturing with astrocytes during chemical induction strongly decreased cell viability and did not improve the neuronal properties (data not shown). The ICFRYA cocktail plus neurotrophic factors and FBS was the best neuronal induction condition. In both BM- and UCB-MSCs, this condition generated neuron-like cells with multiple arborizing dendritic-like structures and high percentages of cells positive for MAP2, NF-H, TAU, and other mature neuron markers such as ENO2, GABA, and NeuN (Figures 3(a) and 3(b)). No significant differences were found between the positive neuron-like cells induced from BM- or UCB-MSCs (Figure 3(c)). BM-MSCs and UCB-MSCs in control conditions did not show reactivity to the neuronal markers, and no cells positive for the glial marker GFAP were detected in the control or induced cultures (Supplementary Figure 3).

**Figure 4:**
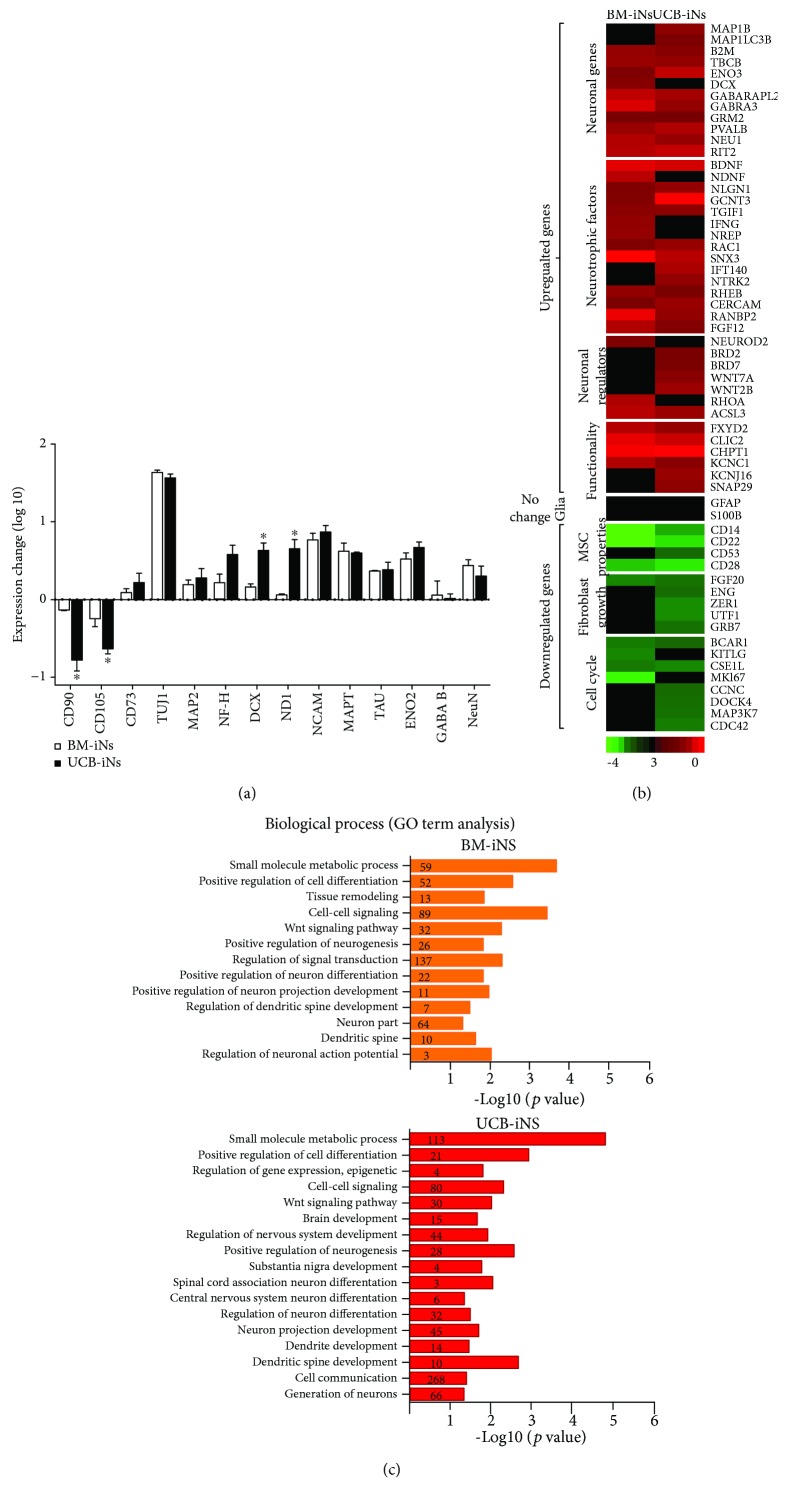
Gene expression analysis in neuron cells induced from UCB-MSCs (UCB-iNs) or BM-MSCs (BM-iNs). (a) Real-time qPCR evaluation of the expression of representative mesenchymal and neuronal genes. Data are shown as fold change versus noninduced MSCs (mean ± SEM, *n* = 3 independent experiments) (^∗^ *p* < 0.05; Student's *t*-test). (b, c) Global gene expression analysis. (b) Heat map showing the change in expression of neuronal, glial, mesenchymal, and cell cycle genes from the microarray data after 8 and 4 days (BM-iNs and UCB-iNs, respectively) of induction by the ICFRYA cocktail plus neurotrophic factors. The fold change in expression was calculated by comparison with noninduced cells from the respective tissue source. Red color indicates increased gene expression while green indicates a decrease. (c) Gene Ontology (GO) analysis demonstrated that in BM-iNs and UCB-iNs, the genes with a ≥1.5-fold change in expression are implicated in the regulation of the neurogenic process. Highlighted in blue are the categories present in both BM- and UCB-INs. The number of genes implicated per category is indicated over the bars.

Changes in the gene expression of neuronal, glial, and MSC markers as detected by qRT-PCR were analyzed after the induction process. The neuron-like cells derived from UCB-MSCs and BM-MSCs showed increased expression of neuronal genes, such as TUJ1, MAP2, NF-H, DCX, NCAM, ND1, NCAM, TAU, ENO2, GABA, and NeuN, and downregulated expression of the MSC markers CD90 and CD105 (Figure 4(a)). The fold change in expression of CD90, CD105, DCX, and ND1 was significantly higher in the cultures generated from UCB-MSCs than from BM-MSC cells.

To evaluate the transdifferentiation process, we conducted a global RNA expression analysis. In the presence of the ICFRYA cocktail, both UCB- and BM-MSC cells upregulated the expression of neuronal regulators and other genes associated with neurodevelopment and functional properties (Figure 4(b)). Among the genes implicated in functional differentiation, the potassium voltage-gated gene KCNC1 was upregulated in BMs, and the KCNC1 and KCNJ16 genes were upregulated in UCB-MSCs. The glial genes GFAP and S100B remained unchanged, and positive regulators of the cell cycle and genes associated with mesenchymal and fibroblastic lineages were downregulated (Figure 4(b)). Gene Ontology term analysis indicated enrichment in the genes related to processes such as small molecule metabolism, cell signal, cell differentiation, positive regulation of neurogenesis, neuronal differentiation, functional neuron activities, dendritic spine development, and action potentials (Figure 4(c)). Altogether, the results indicate that the neuron-like cells obtained can be considered induced neurons (iNs) generated from UCB-MSCs (UCB-iNs) or iNs generated from BM-MSCs (BM-iNs). Although the UCB-iNs and BM-iNs have similar neuronal features, there was a higher number of genes regulated in each Gene Ontology process and a higher *p* value in the UCB-iNs (Figure 4(c)).

### 3.4. Ion Currents in BM-iNs and UCB-iNs

**Figure 5:**
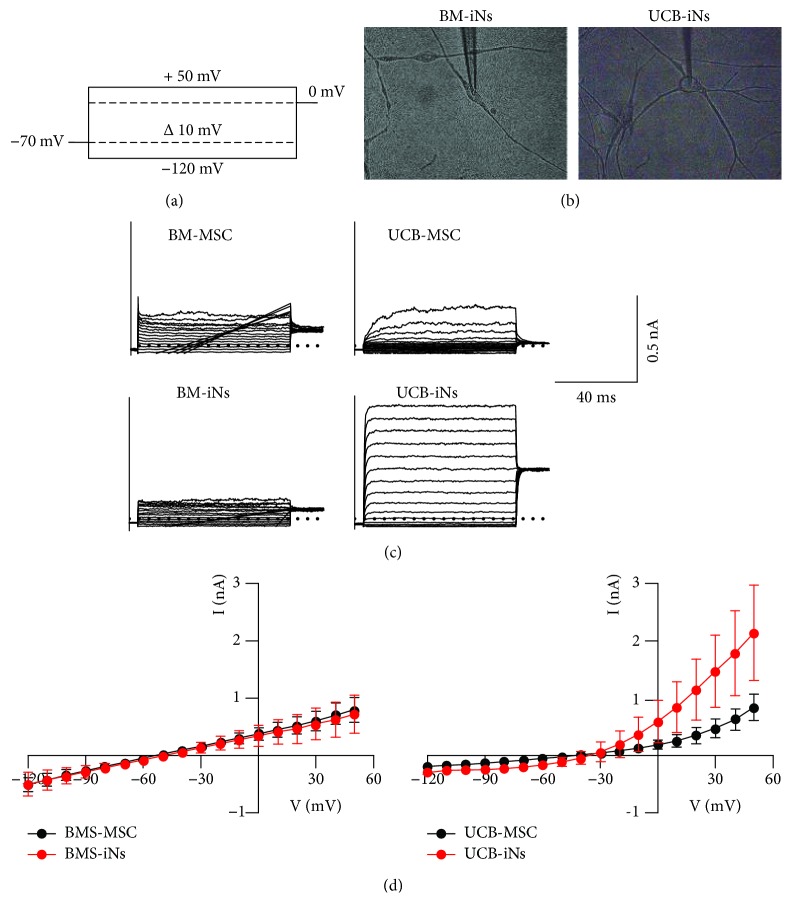
Ion currents in UCB-iNs and BM-iNs: (a) the voltage-clamp protocol used in all experiments (200 ms step pulses from -120 to +50 mV in 10 mV increments from a holding voltage of -70 mV); (b) iN-UCBs or iN-BMs with complex neuron-like morphology were selected for electrophysiological recordings; (c) representative traces of the currents recorded from the control or induced cells. UCB cells presented potassium-like currents, but Na^+^-like currents were not detected. (d) Current-to-voltage relationships. The data are presented as the mean ± SEM.

Whole-cell ion currents were evaluated in control cells (UCB-MSCs and BM-MSCs) and in cells with complex neuron-like morphology from UCB-iNs and BM-iNs (Figures 5(a) and 5(b)). Fast inward sodium-like currents were not detected in the control nor treated conditions. Outward noninactivating ion potassium-like currents were also evaluated. Slight and irregular potassium-like currents were recorded in BM-iNs and no different to those in control BM-MSCs (Figures 5(c) and 5(d)). Potassium-like currents of high magnitude were recorded in 6 of the 14 UCB-iNs, whereas in controls (UCB-MSCs), only small and irregular currents were recorded.

## 4. Discussion

Neuronal transdifferentiation without genetic manipulation from a suitable cell source is the most relevant cell replacement strategy in the brain. This strategy was approached in the present study by generating neuron-like cells from human MSCs with a small molecule cocktail.

The neuronal induction potential of neonatal and adult MSC sources was compared since some studies suggest that MSCs from neonatal sources may have greater plasticity and stemness than adult sources, possibly due to their lower degree of commitment [24, 32, 33]. This hypothesis was not confirmed in the protocol using the ICFRYA small molecule cocktail as the neuronal inductor because the best sources to generate cells with neuronal features were BM-MSCs and UCB-MSCs. In contrast, neuronal traits were not induced from WhJ-MSCs or were limited in the MSCs from dental tissues, SK-MSCs, and PLAC-MSCs.

In addition to the neuronal transdifferentiation potential, other studies have shown that MSCs from different tissues exhibit differences in other functional activities, such as cell proliferation, immune regulation properties, and trilineage differentiation potential [12, 26, 27]. The intrinsic properties of each MSC source or their tissue-specified functions may explain the different responses; however, this has not been demonstrated. Each of the small molecules in the ICFRYA cocktail used to induce neuronal transdifferentiation regulates specific signaling pathways or epigenetic mechanisms [7, 9, 11, 34, 35]. By performing screening assays with small molecule combinations, it is possible to define whether the differences in the neuronal induction efficiency of each specific cocktail are related to the particular signaling pathway or epigenetic mechanism affected by the cocktail molecules in the different MSC sources.

Another relevant feature to be considered in the process of neuronal transdifferentiation is that cell death may function as a regulatory strategy *in vivo* to accomplish successful neurogenesis and neuronal connection in the neural network [36, 37]. During *in vitro* neuronal transdifferentiation, cell death could be a destabilization product of the process itself (activation/inhibition of metabolic pathways), resulting in the elimination of cells that could not adapt to changes. In other studies of neuronal transdifferentiation with small molecule cocktail protocols, cell viability was not examined or presented as a marker of induction efficiency [20, 21]. Here, we evaluated a possible correlation between necrosis and neuronal induction efficiency and found that the ICFRYA cocktail evoked high cell death without neuronal induction in WhJ-MSCs and high neuronal induction efficiency with low cell death in BM- and UCB-MSCs (Figure 2 and Supplement Table 4).

The relevant role of I-BET151 in the neuronal induction by small molecule cocktails has been reported for mouse fibroblasts [22], human astrocytes [20], and in our previous study on BM-MSCs [10]. This effect was extended for all the MSC sources studied here, since removing I-BET151 from the cocktail blocked the presence of neuronal properties regardless of the tissue source. I-BET151 is a regulator that disrupts the epigenetic memory of the original cell, thus favoring cell reprogramming [34, 38–40]. I-BET151 also arrests the cell cycle, a process that is associated with neuronal transdifferentiation [34, 41]; however, I-BET151 alone did not induce neuronal properties. The ICFRYA cocktail contains forskolin and dbcAMP, which increase the levels and synthesis of intracellular cAMP. CHIR99021 inhibits GSK3/beta catenin and promotes the expression of neuronal genes. RepSox is involved in the reprogramming processes that inhibit SMADS, and Y27632 inhibits ROCK to maintain cell viability [7, 35, 42]. Altogether, this suggests that the process of neuronal transdifferentiation requires the synergic regulation of pathways involved in neuronal transdifferentiation, neuronal specification, and survival.

During direct transdifferentiation, the original cells must first undergo partial dedifferentiation, allowing the repression of the lineage of origin and then differentiate into a new cell type [43, 44]; moreover, the cells have to arrest the cell cycle at the same time that the chromatin is modulated [41]. In BM-MSCs and UCB-MSCs, the ICFRYA cocktail enriched with neurotrophic factors (NT3, BDNF, and GDNF) favors the generation of cells with complex neuronal morphology, reactivity to mature neuronal markers, and upregulated neuronal genes and neuronal process. Moreover, mesenchymal markers were decreased, and genes related to mitosis, cell cycle, and mesenchymal lineage were downregulated. Considering all these criteria, the transdifferentiated cells were determined to be induced neurons generated from BM-MSCs (BM-iNs) and UCB-MSCs (UCB-iNs).

To be considered a functional iN, cells should exhibit electrophysiological activity, such as action potentials and synaptic transmission. To examine functional properties, the presence of voltage-gated potassium and sodium currents was evaluated in BM-iNs and UCB-iNs. Sodium currents were not detected, and potassium-like currents were present only in UCB-iNs. These results suggest that UCB-iNs may be in a transition phase towards functionally induced neurons. To obtain functional neurons, it is necessary to test other maturation strategies. Three-dimensional (3D) cultures have been shown to be an option to obtain functionally mature cells during the reprogramming or transdifferentiation processes of several cell types [45]. This option, together with the ICFRYA cocktail, may be a convenient strategy to generate functional iNs from MSCs.

It has been recognized that MSCs present advantages over other cell types and have a low tumorigenic risk in cell replacement therapies. Moreover, clinical studies in human and neurodegenerative animal models indicate that MSCs improve regeneration capacity and ameliorate brain injuries. Although BM-MSCs are commonly considered the “gold standard,” other MSC sources are more accessible and raise fewer ethical issues. Therefore, identifying the most suitable cell type and cell source for neuronal conversion is an essential matter in cell therapy approaches for neurological disorders.

## 5. Conclusion

This study determines that human MSCs can be converted into transgene-free neuronal cells and establishes UCB-MSCs as the most suitable sources for undergoing neuronal transdifferentiation by a specific small molecule cocktail.

## Data Availability

The microarray data used to support the findings of this study have been deposited in the GEO repository (GSE120681).

## Supplementary Materials

Supplementary Materials Supplemental information for this article includes three supplementary figures and five supplementary tables. Supplementary Figure 1, Supplementary Table 1, Supplementary Table 2, and Supplementary Table 3: mesenchymal stem cell isolation and characterization. Supplementary Figure 2 and Supplementary Table 4 are related to Figure 2. Supplementary Figure 3 and Supplementary Table 5 are related to Figure 3.
