# Supplementary material for: Neuronal Transdifferentiation Potential of Human Mesenchymal Stem Cells from Neonatal and Adult Sources by a Small Molecule Cocktail
Source: Stem Cells Int. 2019 Apr 1;2019:7627148. doi: 10.1155/2019/7627148 (PMC6466843; doi:10.1155/2019/7627148)
Supplement: Supplementary Materials — Supplemental information for this article includes three supplementary figures and five supplementary tables. Supplementary Figure 1, Supplementary Table 1, Supplementary Table 2, and Supplementary Table 3: mesenchymal stem cell isolation and characterization. Supplementary Figure 2 and Supplementary Table 4 are related to Figure 2. Supplementary Figure 3 and Supplementary Table 5 are related to Figure 3. [file 7627148.f1.pdf]

SUPPLEMENTARY MATERIAL

Supplementary Table 1. Cell-tissue source information. Three biological replicates were used for each MSC source. Neonatal MSCs were isolated from discarded tissues from the births of 37- to 40-week pregnancies. All samples were obtained from healthy donors and isolated as described in the methods section.

**ADULT SOURCES**						
**MSC SOURCE**	**Biological Replicate 1**		**Biological Replicate 2**		**Biological Replicate 3**	
	AGE	SEX	AGE	SEX	AGE	SEX
DENTAL PULP	17	F	28	F	30	F
PERIODONTAL LIGAMENT	17	F	28	F	30	F
GINGIVAL TISSUE	17	F	28	F	30	F
SKIN	34	M	32	M	35	M
BONE MARROW	25	M	30	M	27	M

**NEONATAL SOURCES (Mother's age)**			
**MSC SOURCE**	**Biological Replicate 1**	**Biological Replicate 2**	**Biological Replicate 3**
WHARTON JELLY	25	26	22
UMBILICAL CORD BLOOD	25	23	27
PLACENTA	32	29	27

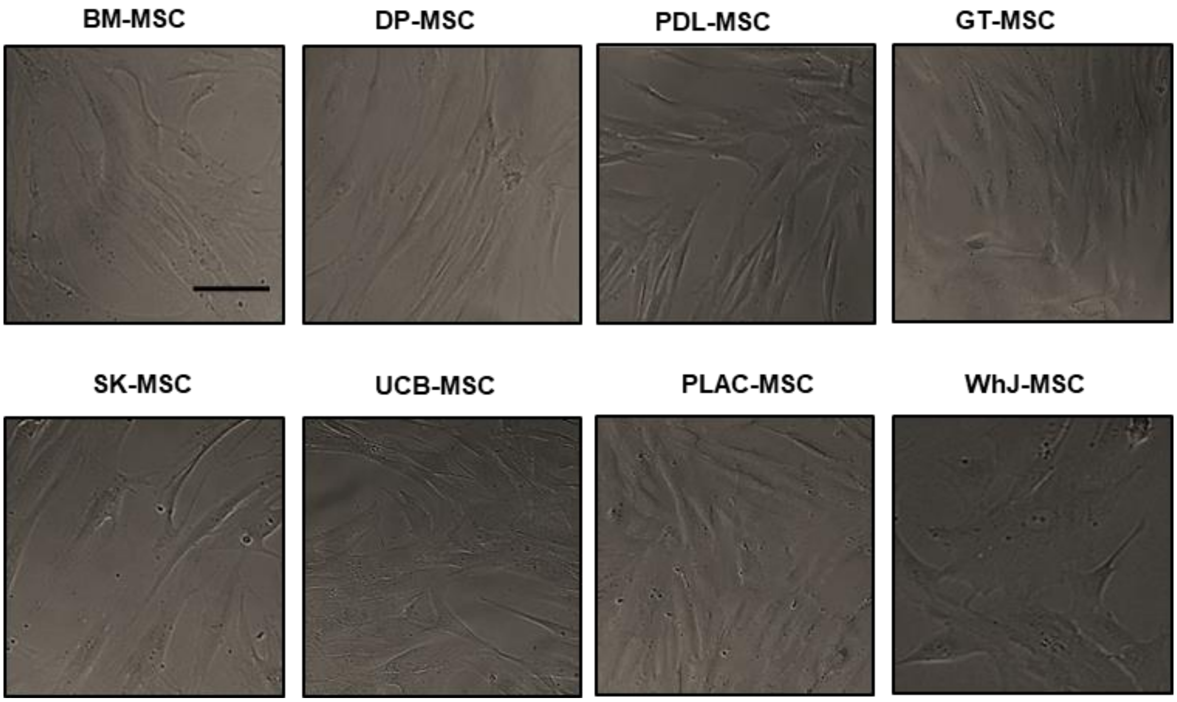

Supplementary Figure 1. Fibroblast morphology of MSC sources. After isolation, MSCs from the different sources presented a typical fibroblastic morphology. Although slight differences in extension or size were found, neuronal-like morphology was not observed in any MSC source. The scale bar represents 100 µm.

Supplementary Table 2. Mesenchymal lineage characterization by membrane markers. All samples present high percentages of mesenchymal markers and low percentages of hematopoietic markers. All data are presented as the mean (n=3) and standard deviation. ND: not detected

SURFACE MARKER	BM-MSC	GT-MSC	DP-MSC	PDL-MSC	SK-MSC	UCB-MSC	PLAC-MSC	WhJ-MSC
CD105	95±8.1	99.7±0.08	92.0±7.6	95.8±3.96	96.4±2.90	91.7±0.88	93.7±0.88	89.3±2.96
CD90	99±0.3	99.7±0.19	99.8±0.13	99.6±0.17	97.2±1.31	77.0±3.51	80.0±5.77	93.3±2.85
CD73	99±0.2	99.8±0.04	98.9±0.32	98.2±1.73	88.8±4.98	95.3±1.45	97.3±0.88	91.7±2.19
CD13	100±0.0	99.8±0.03	96.1±0.283	99.4±0.41	96.6±2.58	96.7±0.88	98.7±0.33	ND
HLA-I	61±22.7	63.0±31.3	53.5±26.5	54.5±28.4	15.0±6.70	87.7±4.33	92.3±1.20	65.7±15.7
HLA-II	3.1±2.7	1.26±0.25	0.11±0.07	1.32±1.22	0.45±0.26	0.67±0.33	0.3±0.33	0.00±0.00
CD45	0.4±0.3	0.06±0.06	0.07±0.05	0.11±0.06	0.99±0.43	0.33±0.33	0.3±0.33	0.33±0.33
CD34	2.9±4.0	0.0±0.0	0.07±0.03	0.05±0.18	1.42±0.68	0.67±0.33	0.7±0.33	0.67±0.67
CD31	0.4±0.4	0.25±0.13	0.26±0.21	0.37±0.07	1.04±0.41	0.00±0.00	0.3±0.33	0.33±0.33
CD14	1.8±2.8	0.02±0.02	0.03±0.05	0.12±0.08	0.81±0.28	0.33±0.33	0.3±0.33	ND

Supplementary Table 3. Mesenchymal characterization by trilineage differentiation. The capacity of differentiation into adipocytes, osteocytes and chondrocytes is represented by crosses, with limited (+) to elevated (+++++) differentiation potential. In SK-MSCS, the potential ranged from limited to middle. ND: not detected, BR: biological replicate

MSCs-SOURCE	ADIPOGENIC			OSTEOGENIC			CHONDROGENIC		
	BR 1	BR 2	BR 3	BR 1	BR 2	BR 3	BR 1	BR 2	BR 3
BM-MSC	+++	++	++	++++	++++	+++	+++++	+++++	+++++
GT-MSC	++	+++	++	++++	++++	+++	+++++	+++++	+++
DP-MSC	+	+	+	++++	++++	+++	+++++	+++++	+++
PDL-MSC	++	++	+	++++	++++	++	+++++	+++++	++
SK-MSC	+	++	+	++	++	N.D.	+	++	+
UCB-MSC	+	+	+	++	++	++	+++	+++	+++
PLAC-MSC	+	+	+	++	++	++	+++	+++	+++
WhJ-MSC	+	+	+	++	++	++	+++	+++	+++

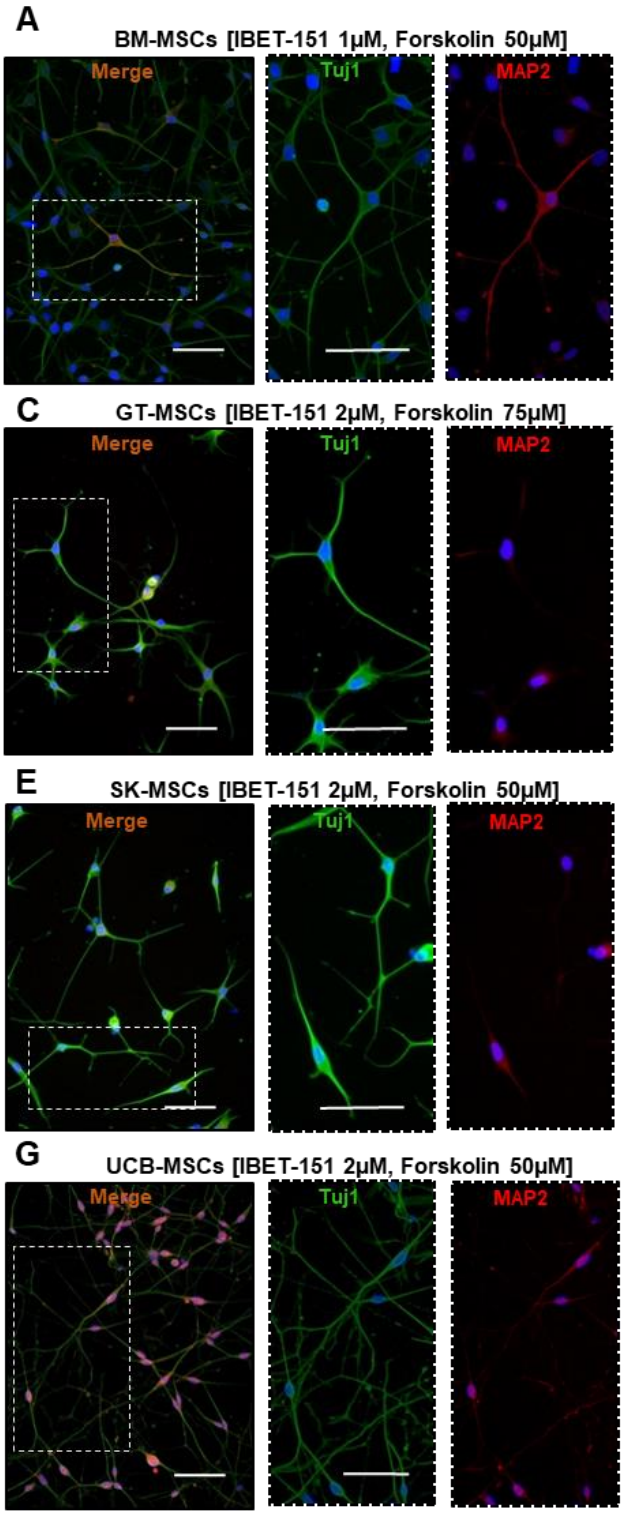

Supplementary Figure 2, related to Figure 2 and Supplementary Table 4. Representative images for TUJ1 and MAP2 reactivity in each MSC source with increasing I-BET and/or forskolin concentration. (A and G) UCB and BM-MSCs were the best sources for neuronal induction. Immunostaining showed cells positive for TUJ1 and MAP2 marker branched associated in both complex bodies. (B – E and H) Sources with intermediate effects. Immunostaining showed low numbers of cells per field, some with neuron-like morphology and MAP2 slight reactivity close to the nucleus and not in the branches. (F) Wharton's Jelly cells showed no neuronal induction. Representative images of three biological replicates per source. The scale bar represents 100 µm.

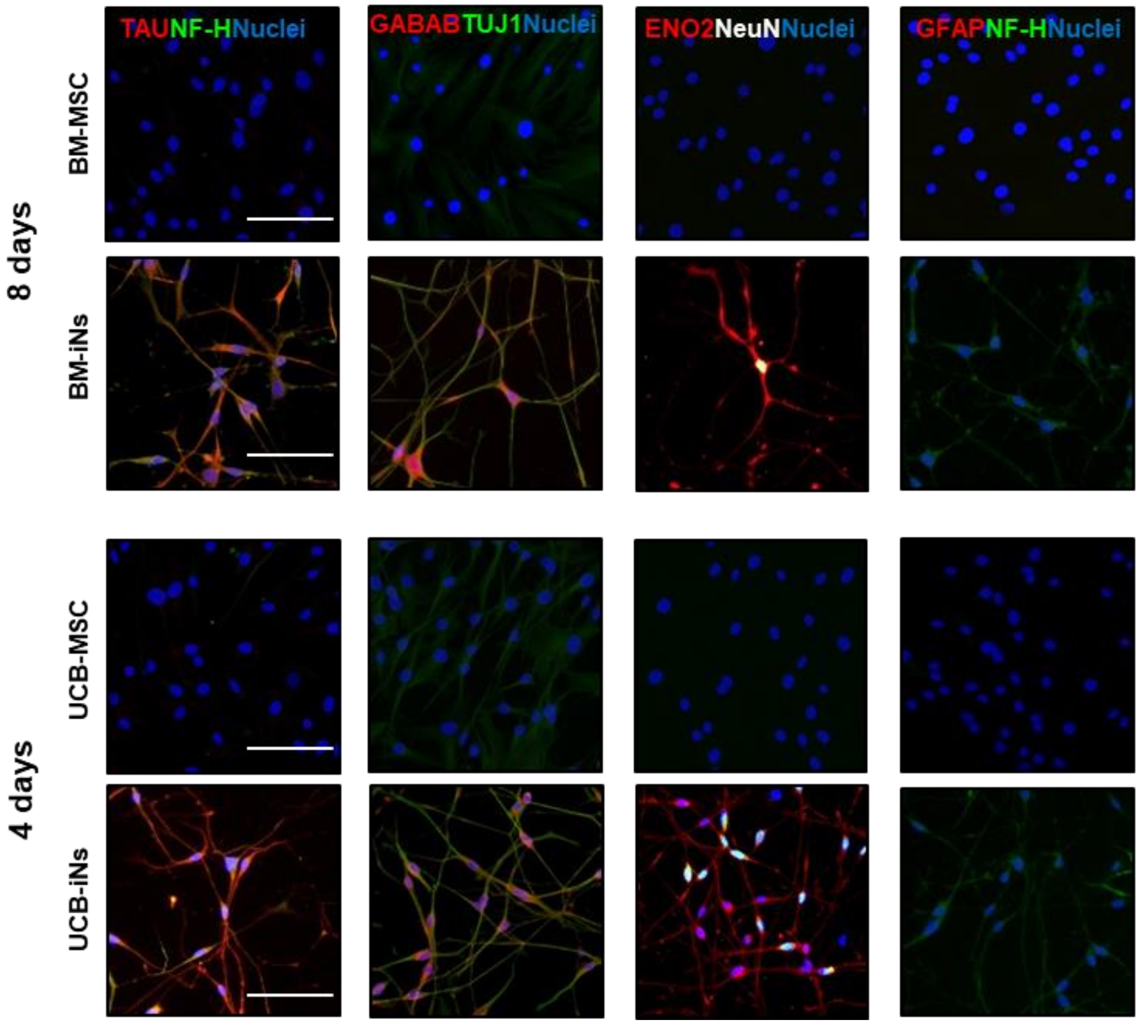

Supplementary Figure 3, related to Figure 2. Mature neuronal marker reactivity in control and induced BM-MSCs and UCB-MSCS. (A and B) Immunostaining analysis of neuronal markers (TAU, NF-H, GABA B, TUJ1, ENO2, NeuN) and astrocyte marker (GFAP) in MSCs cultured only in NM for 8 or 4 days and after the neuronal induction process. The GFAP marker was negative in cells under basal conditions and in the process of differentiation, while the neuronal mature markers only appear in BM-iNs and UCB-iNs. The TUJ1 neuronal marker has basal reactivity in undifferentiated MSCs, which was considered negative due to the absence of neuronal morphology. Representative images of n=3 independent experiments are shown. The scale bar represents 100 µm.

Supplementary Table 4, related to Figures 1 and 2. Percentages of neuronal properties and necrosis in all sources of MSCs with increasing I-BET151 and/or forskolin concentrations. The table shows the change in neuronal properties and necrosis generated by the original ICFRYA cocktail, with the elimination of I-BET151 and with increasing concentrations of I-BET151 and/or forskolin, from which the best conditions for each source were selected.

CONDITION<br>PROPERTIE	ICFRYA	NO IBET-151	I [2µM]	F [75µM]	I [2µM]<br>F [75µM]
**WhJ-MSC (8 days)**					
**TUJ1**	0.1±0.3	0.9±1.2	1.3±1.5	0.9±0.6	3.7±3.6
**MAP2**	0.0±0.0	0.0±0.0	0.1±0.3	0.0±0.0	0.0±
**Necrosis**	48.3±10.8	43.3±23.5	63.4±13.5	46.2±19.6	68.2±8.8
**DP-MSC (8 days)**					
**TUJ1**	49.5±13.4	12.3±10.9	49.5±18.7	43.8±11.6	62.3±15.2
**MAP2**	0.7±0.9	0.1±0.2	4.4±6.7	1.9±3.0	6.1±12.5
**Necrosis**	50.7±11.8	40.9±19.6	64.1±4.8	58.2±7.8	70.6±8.8
**PDL-MSC (8 days)**					
**TUJ1**	42.1±12.1	11.7±9.5	49.5±20.2	47.3±14.8	54.7±20.4
**MAP2**	2.2±2.6	0.0±0.0	10.3±10.8	11.2±13.5	12.4±8.4
**Necrosis**	62.0±12.0	34.9±21.1	68.3±12.0	72.7±13.9	82.6±9.6
**GT-MSC (8 days)**					
**TUJ1**	31.8±12.7	3.5±3.7	29.2±10.5	32.5±13.7	23.8±1.4
**MAP2**	2.0±2.5	0.0±0.0	3.4±5.0	3.5±4.0	1.2±2.1
**Necrosis**	52.1±15.0	18.8±14.0	58.3±16.9	64.6±13.7	81.8±9.5
**SK-MSC (8 days)**					
**TUJ1**	34.1±11.7	0.1±0.1	56.4±36.7	41.5±27.0	50.3±32.6
**MAP2**	6.3±5.8	0.0±0.0	8.4±6.0	2.2±1.1	6.3±4.8
**Necrosis**	68.6±22.7	3.0±1.3	71.7±11.5	74.2±18.0	80.4±10.3
**PLAC-MSC (8 days)**					
**TUJ1**	67.2±4.0	1.9±1.0	71.0±2.7	68.5±6.5	ND
**MAP2**	1.8±1.0	0.0±0.0	2.3±0.4	1.7±1.5	ND
**Necrosis**	53.4±2.0	35.1±18.9	63.7±8.9	56.6±10.7	ND
**UCB-MSC (4 days)**					
**TUJ1**	80.7±9.7	0.9±0.9	77.6±10.7	75.1±11.9	ND
**MAP2**	1.9±0.5	0.0±0.0	4.9±0.4	1.8±0.6	ND
**Necrosis**	17.9±10.3	3.2±1.7	25.0±8.8	22.8±9.8	ND
**BM-MSC (8 days)**					
**TUJ1**	53.9±12.0	2.9±1.2	66.2±10.3	56.3±8.5	41.5±18.6
**MAP2**	12.4±4.0	0.0±0.0	24.5±4.7	16.0±4.0	14.3±5.8
**Necrosis**	48.6±6.3	8.9±7.8	71.8±5.5	64.7±13.8	75.9±11.2

Supplementary Table 5, related to figure 4. Primers for qPCR used in this study.

PCR PRIMERS	REFSEQ	FORWARD (5' - 3')	REVERSE (5' - 3')	PRODUCT SIZE (bp)	AMPLIFICATION EFFICIENCY
TATA box binding protein (TBP)	NM_003194.4	GCAAGGGTTTCTGGTTTGCC	CAAGCCCTGAGCGTAAGGTG	80	1.05
Hypoxanthine phosphoribosyl transferase 1 (HPRT1)	NM_000194.2	CCCTGGCGTCGTGATTAGTG	TCGAGCAAGACGTTCAGTCC	139	1.01
Thy-1 cell surface antigen (THY1) (CD90)	NM_006288.3	CAGCATCGCTCTCCTGCTAA	ACTGGATGGGTGAACTGCTG	134	0.99
Endoglin (ENG) (CD105)	NM_00018.3	CCTGACCTGTCTGGTTGCAC	ACGCGTGTGCGAGTAGATG	126	1.04
5' ecto-nucleotidase (CD73) (NT5E)	NM_002526.3	GATGAACGCAACAATGGCACA	CAAATGTGCCTCCAAAGGGC	70	1.09
Tubulin, beta 3 class III (βIIITUB)	NM_006086.3	AGTGCGGCAACCAGATCG	GCACGTACTTGTGAGAAGAGG	150	1.04
Microtubule-associated protein 2 (MAP2)	NM_002374.3	GCTCCCGGAGAAGGATTCTG	CAAGCTGAAGAATCAGCGCA	82	0.91
Neurofilament, heavy polypeptide (NF-H)	NM_021076.3	GAGGAGTGGTTCCGAGTGAG	CGTCTGTGTTCACCTTGGCT	61	1.06
Doublecortin (DCX)	NM_000555.3	AAAGACACTGGCTGTTCCCTG	CCCAAGGTTAGCATCTCCAGC	184	1.02
Neuronal differentiation 1 (NEUROD1)	NM_002500.4	ACAACAAAGGAAATCGAAACATGAC	ATCAGCCCACTCTCGCTGTA	52	1.02
Neural Cell Adhesion Molecule (NCAM)	NM_000615.6	GCAGCGAAGAAAAGACTCTGG	CTGGATGCTCTTCAGGGTCA	83	1.04
Microtubule associated protein Tau (MAPT)	NM_016835.4	ACCAGCTCCGGCACCAA	CCTGGTTCAAAGTTCACCTGATAG	134	1.03
Microtubule associated protein Tau (TAU)	NM_016835.4	TCCGGCACCAACAGCAG	TCCTGGTTCAAAGTTCACCTGAT	129	1.03
Enolase 2 (ENO2)	NM_001975.2	TGCACAGGCCAGATCAAGAC	ACAGCACACTGGGATTACGG	139	0.99
Gamma-aminobutyric acid type B receptor subunit 1 (GABAB)	NM_001319053.1	ACTGTTTCCCATGAGCGGG	TGGATCACACTTGCTGTCGT	145	1.05
RNA binding protein, fox-1 homolog (C. elegans) 3 (RBFOX3) (NeuN)	NM_001082575.2	AGCCTACAGCGACAGTTACG	GTCCGAGAAGGAAACGGTGG	123	1.1
